# Enhanced Arsenate Removal Performance in Aqueous Solution by Yttrium-Based Adsorbents

**DOI:** 10.3390/ijerph121013523

**Published:** 2015-10-26

**Authors:** Sang-Ho Lee, Kyoung-Woong Kim, Byung-Tae Lee, Sunbaek Bang, Hyunseok Kim, Hyorang Kang, Am Jang

**Affiliations:** 1 School of Environmental Science and Engineering, Gwangju Institute of Science and Technology, 123, Cheomdangwagi-ro, Buk-gu, Gwangju 61005, Korea; E-Mails: ddlee19@gist.ac.kr (S.-H.L.); btlee@gist.ac.kr (B.-T.L.); 2 Mine Reclamation Corporation, 2, Segye-ro, Wonju-si, Gangwon-do 26464, Korea; E-Mail: sbang@mireco.or.kr; 3 Energy Lab, Samsung Advanced Institute of Technology, 130 Samsung-ro, Yeongtong-gu, Suwon-si, Gyeonggi-do 16678, Korea; E-Mails: hs13.kim@samsung.com (H.K.); hr12.kang@samsung.com (H.K.); 4 School of Civil and Environmental Engineering, Sungkyunkwan University, 2066, Seobu-ro, Jangan-gu, Suwon-si, Gyeonggi-do 16419, Korea; E-Mail: amjang68@gmail.com

**Keywords:** adsorption, arsenate removal, basic yttrium carbonate (BYC), surface modification, specific surface area, maximum adsorption capacity

## Abstract

Arsenic contamination in drinking water has become an increasingly important issue due to its high toxicity to humans. The present study focuses on the development of the yttrium-based adsorbents, with basic yttrium carbonate (BYC), Ti-loaded basic yttrium carbonate (Ti-loaded BYC) and yttrium hydroxide prepared using a co-precipitation method. The Langmuir isotherm results confirmed the maximum adsorption capacity of Ti-loaded BYC (348.5 mg/g) was 25% higher than either BYC (289.6 mg/g) or yttrium hydroxide (206.5 mg/g) due to its increased specific surface area (82 m^2^/g) and surface charge (PZC: 8.4). Pseudo first- and second-order kinetic models further confirmed that the arsenate removal rate of Ti-loaded BYC was faster than for BYC and yttrium hydroxide. It was subsequently posited that the dominant removal mechanism of BYC and Ti-loaded BYC was the carbonate-arsenate ion exchange process, whereas yttrium hydroxide was regarded to be a co-precipitation process. The Ti-loaded BYC also displayed the highest adsorption affinity for a wide pH range (3–11) and in the presence of coexisting anionic species such as phosphate, silicate, and bicarbonate. Therefore, it is expected that Ti-loaded BYC can be used as an effective and practical adsorbent for arsenate remediation in drinking water.

## 1. Introduction

Arsenic is a well-known strong carcinogen and toxic element [1]. The contamination of arsenic in drinking water has increasingly become an emerging environmental issue due to its strong toxicity and global distribution [2,3]. Several studies have reported that arsenic can cause serious skin and internal cancers, especially in the liver, bladder, and kidneys [4]. In particular, severe arsenic contamination levels and hazardous effects on humans have continuously reported in South-East Asian countries such as Vietnam, Lao PDR and Cambodia [5,6,7]. In order to reduce these noxious human health effects, the World Health Organization (WHO) and United States Environmental Protection Agency (USEPA) have recommended a maximum arsenic concentration of 10 ppb (μg/L) of arsenic as a drinking water standard [8,9], and have discussed that this standard should actually be reduced to 5 ppb[10]. Since this strict drinking water criteria has been applied, high-efficiency and cost-effective technologies have been needed to treat and remove arsenic from drinking water [11].

In the attempts to remove arsenic, the USEPA has promoted four representative techniques for practical arsenic removal: membrane, ion-exchange, coagulation, and adsorption techniques. Of these, adsorption is a process that uses substances to attract and hold pollutants on their surface [12]; it has been widely used to remove arsenic due to its cost-effectiveness, ease of installation, and convenience of management. The lifetime of adsorption systems is strongly dependent on the capacity of the adsorbent and iron and aluminum-based materials have been developed and applied to enhance the adsorbent systems [12,13,14]. Iron and aluminum based adsorbents such as ferrihydrite, ferric oxide and hydroxide, goethite, hematite, activated alumina, and layered double hydroxides have been reported in water remediation [15,16,17]. However, these adsorbents have a common problem of low adsorption capacity, therefore frequent replacement is required during long-term treatments [11]. Hence, a high capacity adsorbent should be developed in order to maintain sustainable water purification systems.

In general, the mechanism of arsenic adsorption is based on the combination of arsenic with metal oxides and hydroxides based on their covalent bonding with oxygen. A previous study [18] has also shown that the hydroxyl group on the adsorbent surface is the key factor for determining the adsorption efficiency as the atomic structure of the surface produces an attraction for arsenic—with the large surface area being more favorable for arsenic adsorption [19]. To increase the surface effectiveness, various modification methods have been applied, and previous studies demonstrated that porous and mesoporous materials display a relatively high removal efficiency for arsenic adsorption, by increasing the specific surface area due to the formation of internal pores [20,21]. Gupta *et al.* [22] and Zhang *et al.* [23] also demonstrated that the adsorption capacity of the binary oxide can be dramatically increased by increasing its specific surface area and hydroxyl groups. Lee *et al*. [24] represented enhanced arsenic adsorption capacity of hybridized layered double hydroxide by thermal treatment which increases surface area and sorption sites. Other studies have reported that the addition of tetravalent ions such as Zr^4+^ and Ti^4+^ in adsorbents increase the adsorption capacity of arsenate and phosphate ions by the increasing surface charge by forming hydroxides and oxides [25,26], as it is possible to modify the surface characteristics of these adsorbents into nanostructured materials by using the co-precipitation method.

In recent years, several arsenic adsorbents based on rare-earth metals using zirconium, cerium, and yttrium have been reported in order to develop novel adsorbents that have a high adsorption capacity [27,28,29]. These rare-earth based adsorbents can be successfully applied to contaminated areas and display superior performance for removing arsenic. In this study, we focused on the synthesis of rare earth metal (Y; yttrium) based arsenic adsorbents by the simple surface modification method. We introduced basic yttrium carbonate (BYC), titanium (Ti) loaded BYC and yttrium hydroxide with high adsorption capacity for arsenate. We investigated the adsorption mechanism of adsorbents by structural characterization using transmission electron microscopy (TEM) and Fourier-transform infrared spectroscopy (FTIR). We also studied the pH effect, adsorption kinetics, isotherm, effect of competitive anions, and regeneration tests to better understand the adsorption behavior in potential remediation applications of arsenic-contaminated water by yttrium based adsorbents.

## 2. Materials and Methods

All chemicals were analytical grade and artificial drinking water was prepared using International Electrical Commission Standards [30]. Synthesized arsenic contaminated water was prepared by spiking the stock solution.

### 2.1. Synthesis of Yttrium-Based Adsorbents

Basic yttrium carbonate (BYC) was synthesized following the method established by Wassay *et al.* [31]. The solutions were mixed to obtain the pH in the range of 6.5 to 7 at about 95 °C under agitation for 1 h. The precipitates were washed to remove excessive sodium ion until the electric conductivity of the washed solution became lower than 15 µS/cm and the final precipitate was then dried at 105 °C for 24 h in dry oven. The Ti-loaded yttrium carbonate was synthesized using YCl_3_·6H_2_O (0.2 M), NH_2_CONH_2_ (0.5 M), and Ti_2_(SO_4_)_2_ (0.2 M) (Alfa Aesar, Ward Hill, MA, USA). YCl_3_·6H_2_O (0.2 M) and Ti_2_(SO_4_)_2_ (0.2 M) were mixed at different ratios and each precipitate was treated using the same procedure as for BYC. In the case of yttrium hydroxide, 1 M NaOH was continuously added to the 0.2 M YCl_3_ solution until pH 7.0 was attained. The precipitate was then dried at 105 °C in an oven and ground through a 100 mesh sieve. The powder samples were finally stored in a desiccator prior to further analyses.

### 2.2. Adsorbent Characterization

In order to characterize the synthesized adsorbents, X-ray diffraction (XRD; SIEMENS D5005 Diffractometer, Karlsruhe, Germany) was used to identify differences in the crystalline structures of the adsorbents. Fourier-transform infrared spectroscopy (FT-IR; Jasco FT-IR 460 Plus, Tokyo, Japan) analysis was carried out to differentiate the functional groups of the adsorbents based on the change of spectra. Transmission electron microscopy and energy dispersive spectroscopy (TEM-EDS; JEOL JEM-2100, Tokyo Japan) analyses were then carried out to identify variations in the morphology and elemental ratio, and the Brunauer-Emmet-Teller (BET; Micromeritics ASAP 2010 analyzer, Norcross, GA, USA) analysis was conducted to determine the surface area and pore characteristics of the synthesized adsorbents.

### 2.3. Adsorption, Effect of Coexisting Anionic Species, Desorption and Regeneration Tests

Simple arsenic adsorption experiments using BYC with commercial adsorbents were conducted using zero-valent iron (U.S. Metals, Mentone, IN, USA), activated alumina (Alcan Inc., Shawinigan, QC, Canada), iron-coated activated alumina (Alcan Inc.), iron oxide (Sigma Aldrich, St Louis, MO, USA), and granular ferric oxide (Apyron Technologies, Atlanta, GA, USA). The adsorption experiments for yttrium-based adsorbents were carried out at different pH values. The pH range of the initial solution was adjusted from 3 to 11, with the pH maintained using 1 M HCl and NaOH (Sigma Aldrich), as needed. The initial concentration of arsenate was adjusted to 50 mg/L, at a dosage of 1 g/L and reaction time of 24 h. The temperature was maintained at 25 °C in the shaking incubator (HB-201SF, Hanbaek Science, Bucheon, Korea). Kinetic removal experiments using yttrium-based adsorbents were conducted using 50 mg/L arsenate solution to determine the removal rate at pH 7. In the isothermal experiments, the initial arsenate concentrations were adjusted from 10 to 1000 mg/L and the experiments were conducted at pH 4, 7 and 10 for each experimental set.

The effect of coexisting anionic species was determined by comparing the effects of phosphate, silicate, and bicarbonate. Each anion solution was prepared using NaH_2_PO_4_, Na_2_SiO_3_, and NaHCO_3_ (Sigma Aldrich). To compare the effects of coexisting anionic species, 10 mg/L arsenate-spiked water was prepared and the same concentrations of anion solution (10 mg/L) was added into the arsenate solution. The results were subsequently compared to the relative ratio based on the arsenate removal. Desorption experiments were carried out to determine the stability between adsorbent and arsenate. Initially, 0.05 g of the yttrium-based adsorbent was saturated with excessive arsenate solution, and the used adsorbent was then filtered and dried in the oven for 12 h before being reacted in 50 ml of different pH solutions (3 to 13) for 24 h. The regeneration experiments were conducted by repeating the adsorption (at pH 7) and desorption (at pH 13) experiments. After adsorption, the mixture was centrifuged to separate solution, then the basic solution was added to desorb arsenate ion from the adsorbents. At the end of desorption procedure, the basic solution was removed and the arsenic solution was added again with pH adjustment. All samples were filtered using a 0.45 µm membrane filter and subsequently analyzed by inductively coupled plasma-optical emission spectroscopy (ICP-OES; Perkin Elmer 5400DV, Wellesley, Ma, USA).

## 3. Results and Discussion

### 3.1. Optimization of the Ti-loaded BYC

**Figure 1:**
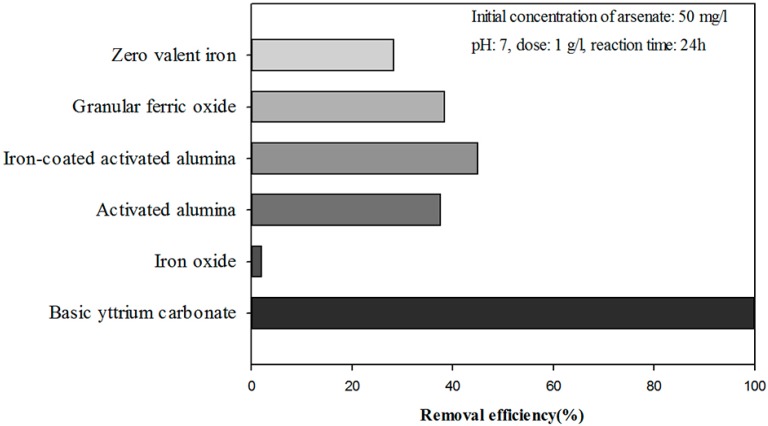
Batch result of basic yttrium carbonate and commercial arsenic adsorbents.

Batch tests were conducted to examine the adsorption capability of arsenate using BYC and commercial adsorbents such as zero-valent iron, granular ferric oxide, activated alumina, iron-coated activated alumina, and iron oxide. Subsequently, BYC demonstrated the best removal efficiency in a 50 mg/L arsenate solution at equilibrium states (Figure 1). Based on the results of Figure 1, BYC was selected as the precursor to synthesize a new adsorbent, as it had the highest adsorption capacity. To modify the BYC, titanium was loaded using a doping method due to its tendency to form an internal chemical complex of titanium dioxide [32,33].

**Figure 2:**
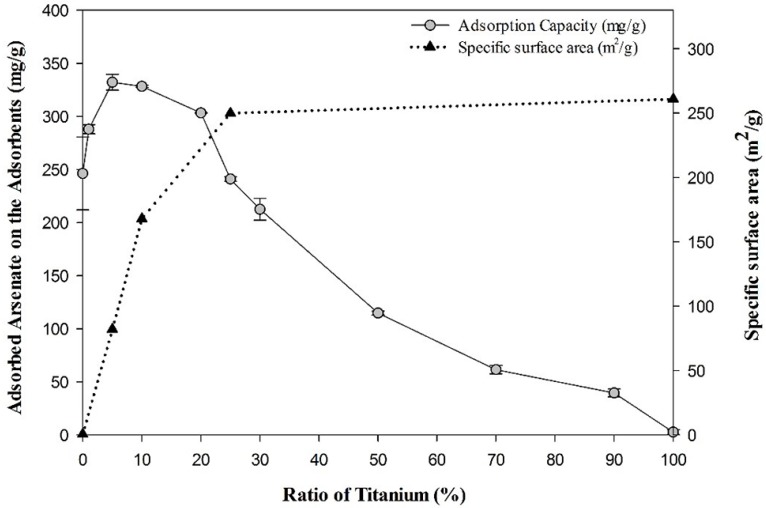
Synergistic effect of arsenate adsorption by Ti-loaded BYC in accordance with the variation of titanium ratio in BYC (Initial concentration of arsenate: 1000 mg/L; pH: 7; Reaction time: 24 h; Temperature: 298K; titanium ratio: atomic ratio).

For the identification of the optimum production conditions of the Ti-loaded BYC, batch tests were conducted by varying the titanium ratio in BYC. It was found that the adsorption capacity of Ti-loaded BYC increased until the titanium ratio reached 5%, with the increase of specific surface area and titanium ratio. The specific surface area of BYC obtained by BET was 0.6 m^2^/g, though the surface area of 5% Ti-loaded BYC was 82 m^2^/g (Figure 2). In addition, the increases in surface area reached the maximum state at the titanium ratio load of 25%, with the adsorption capacity decreasing after exceeding the optimum ratio of Ti ions because the relative ratio of BYC decreased.

### 3.2. Determination of Surface Characteristics in Yttrium-based Adsorbents 

**Figure 3:**
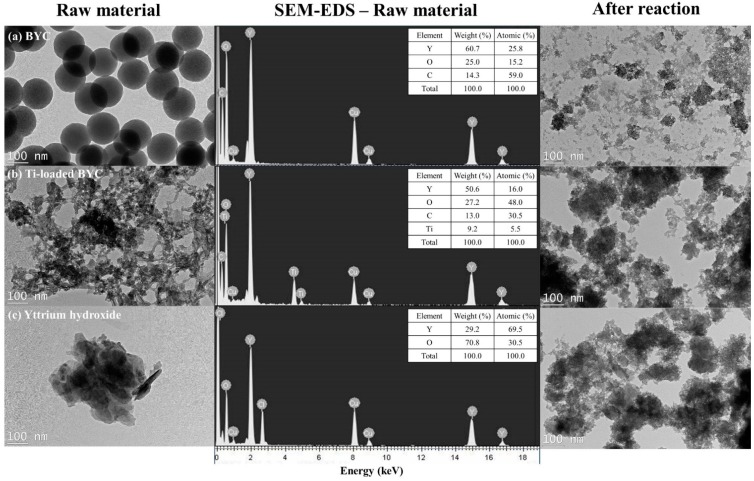
Transmission Electron Microscope images and EDS spectrum of BYC (**a**), 5% Ti-loaded BYC (**b**), and yttrium hydroxide (**c**).

In order to identify the surface characteristics of yttrium-based adsorbents, the surface was examined by TEM-EDS because XRD is unable to determine crystallographic differences among the samples that show the amorphous crystalline phase. In addition, the cause of the no crystalline phase of titanium dioxide in all samples is that the titanium ratio (5%) was not sufficient to be detected by XRD and that the crystalline structure of TiO_2_ such as anatase and rutile did not form due to the low production temperature and lack of thermal treatment [34]. TEM data revealed that BYC displayed the spherical structure (size: 120–160 nm; Figure 3a) due to the formation of carbonate groups via urea hydrolysis; the previous study reporting that the formation of carbonate groups by urea hydrolysis can form a uniform spherical precipitate [35,36]. However, the Ti-loaded BYC has the relatively small particle size with dimensions of about 10 nm to 30 nm (Figure 3b) because the morphological change is related to the formation of binary oxide with titanium and BYC. 

**Figure 4:**
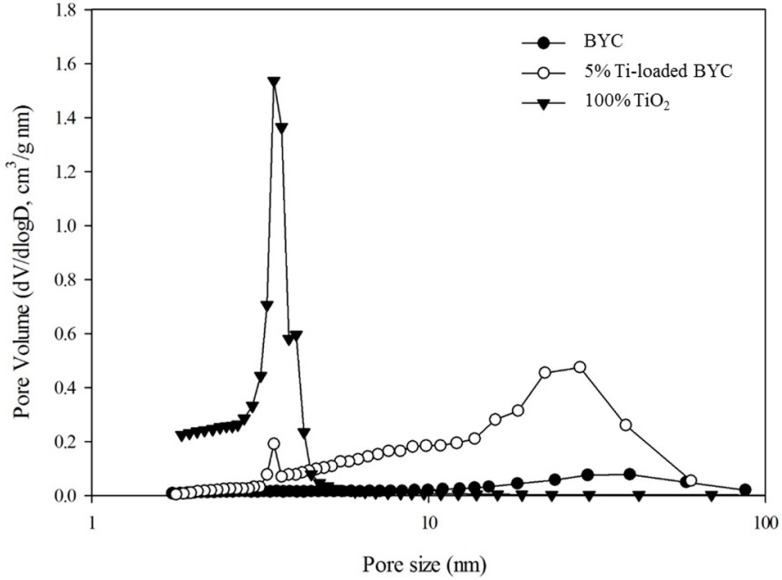
Pore size distribution of yttrium based adsorbents and titanium dioxide by BET analysis.

This increase in the specific surface area was confirmed by the pore size distribution analysis, with the main cause of adsorption enhancement subsequently defined as the formation of nanosized pores by binary oxide of titanium and BYC, and the dominant pore size of between 10 nm and 100 nm (Figure 4). Yttrium hydroxide displayed the multiple layers of thin films due to the formation of a precipitate by NaOH titration (Figure 3c), and also had the specific surface area below 0.1 m^2^/g. The spherical structure of BYC became destroyed due to the reaction between arsenate and carbonate exchange process, and morphological changes of Ti-loaded BYC and yttrium hydroxide were also observed (Figure 3).

The measured points of zero charge (PZC) of BYC, Ti-loaded BYC, and yttrium hydroxide were 7.9, 8.4 and 10.3, respectively. With the addition of titanium into the adsorbents, the removal capacity was enhanced by the increased electrostatic attraction [11] The arsenic species (HAsO_4_^2−^) is present as a strong electron donor, and it can strongly interact with the centered Y^3+^ in the Y(OH)CO_3_ framework via an ion exchange with competing carbonate species. Therefore, Ti-loaded BYC can adsorb more arsenate ions with increasing specific surface area and surface charge. Although the PZC of yttrium hydroxide was higher than that of either BYC or Ti-loaded BYC, its removal efficiency was lower; there may be dominant effect of co-precipitation process rather than the chemical adsorption process by carbonate-arsenate ion exchange process in yttrium hydroxide. Table 1 summarizes the comparison of maximum adsorption capacities for arsenate and physical properties for different adsorbents. Ti-loaded BYC has the highest arsenic adsorption capacity (348.5 mg/g) and it can be deduced from the synergistic effect of increased surface charge and specific surface area even though other materials have higher specific surface area and surface charge (Table 1). Consequently, the Ti-loaded BYC is expected to be advantageous to adsorb arsenate ions by physical properties such as enhanced specific surface area and positive surface charge.

**Table 1:** Comparison of maximum adsorption capacities for arsenate and physical properties for different adsorbents.

Adsorbent	PZC *	Specific Surface Area (m^2^/g)	pH	Maximum Adsorption Capacity (mg/g)	References
BYC	7.9	<1	7.0	289.6	This study
Ti-loaded BYC	8.4	82	7.0	348.5	This study
Yttrium hydroxide	10.3	<1	7.0	206.5	This study
Magnetite	-	179	7.0	16.6	[37]
Akaganeite	-	111	-	29.0	[38]
Goethite	6.7	39	-	4.0	[19]
Fe-Cu binary oxide	7.9	282	7	82.7	[39]
CuO nanoparticles	9.4	21	8	22.6	[40]
Mesoporous alumina	-	312	6.6	36.6	[41]
Nano TiO_2_	5.8	329	7.0	37.5	[42]
CeO_2_-ZrO_2_	-	29	6.9	145.4	[28]
Y-Mn binary composite	7.1	-	7.0	279.9	[29]
Zr nanoparticles	2.9	-	3.0	256.4	[27]

Note: * PZC: Point of zero charge.

### 3.3. FT-IR Study for the Measurement of the Functional Groups

**Figure 5:**
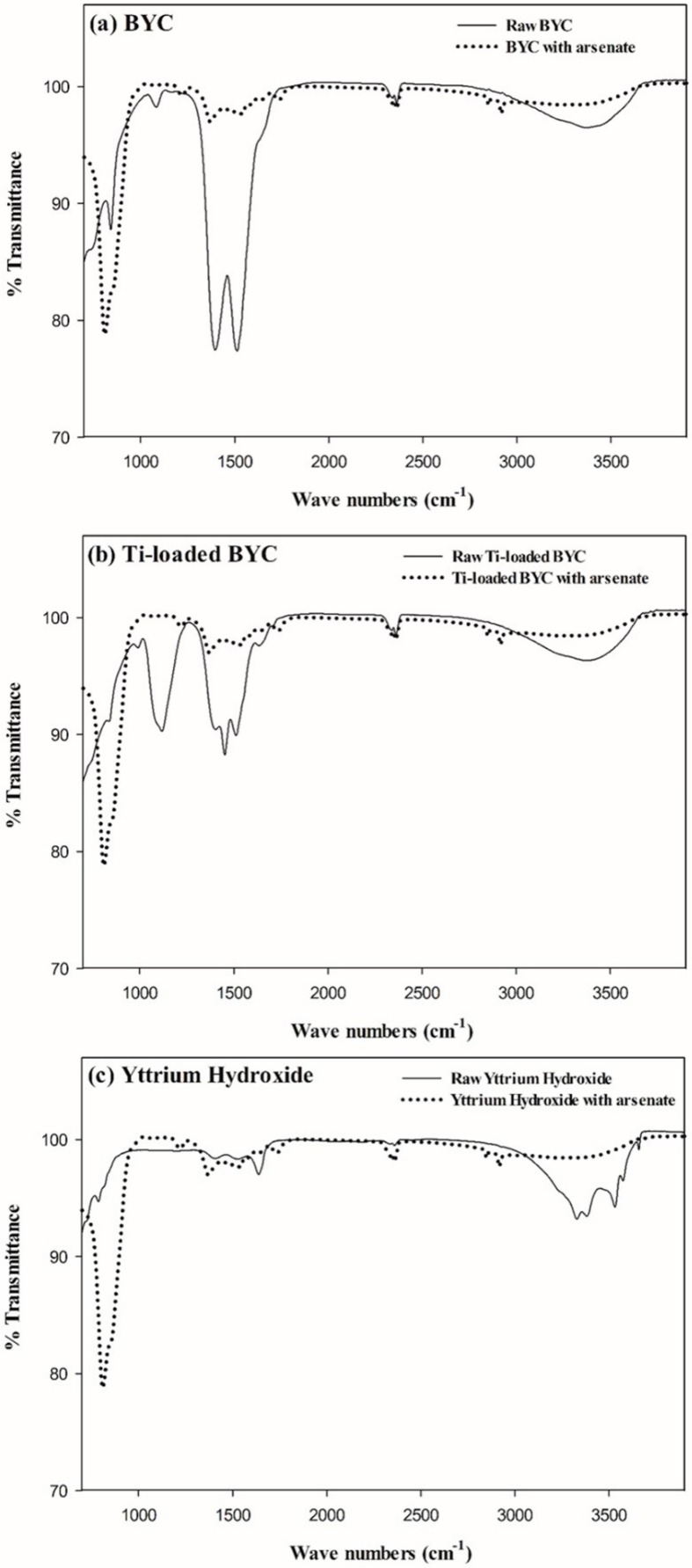
Variation of Fourier Transform IR spectra in BYC (**a**), Ti-loaded BYC (**b**), and yttrium hydroxide (**c**) by reaction with arsenate (black line: raw materials, dotted line: reaction with arsenate).

In order to determine how the functional groups were influenced by the reaction with arsenate ions, variations in the functional groups on the adsorbent surfaces were measured using FT-IR. Figure 5 presents the variation of spectra between the raw materials and materials reacted with arsenate. The band at 3400–3450 cm^−1^ is assigned to the vibration of O–H stretching in all adsorbents [43]. Figures 5a,b present bands at 1280 cm^−1^ to 1420 cm^−1^, which are assigned to the asymmetric stretching vibration of carbonate spectra comprised of aliphatic C=O and C–O [44]. In the BYC and Ti-loaded BYC, O–H and carbonate bands were observed, whereas yttrium hydroxide showed only the O–H band. In samples reacted with arsenate, the bands at hydroxyl (3400 cm^−1^) decreased and the carbonate (1420 cm^−1^) bands disappeared, compared to the raw materials. After adsorption, the new band at 813 cm^−1^ in all materials was observed. The new bands were attributed to As-O vibration of arsenate ion [45]. The equilibrium pH slightly increased to 7.6 after adsorption, with the carbonate-arsenate ion exchange being dominant for the removal process in the case of BYC [31]. However, yttrium hydroxide can remove arsenic by the co-precipitation pathway. Therefore, the reaction for the removal mechanism is assumed to be as follows:

(1) Y(OH)CO_3_ + 2HAsO_4_^2−^ → Y(OH)(HAsO_4_)_2_ + CO_3_^2−^ (Chemical adsorption)

(2) Y(OH)_3_ + 3HAsO_4_^2−^ → Y(HAsO_4_)_3_ + 3H_2_O (Co-precipitation)

### 3.4. Arsenate Removal by the Yttrium-based Adsorbents and Adsorption Isotherm

**Figure 6:**
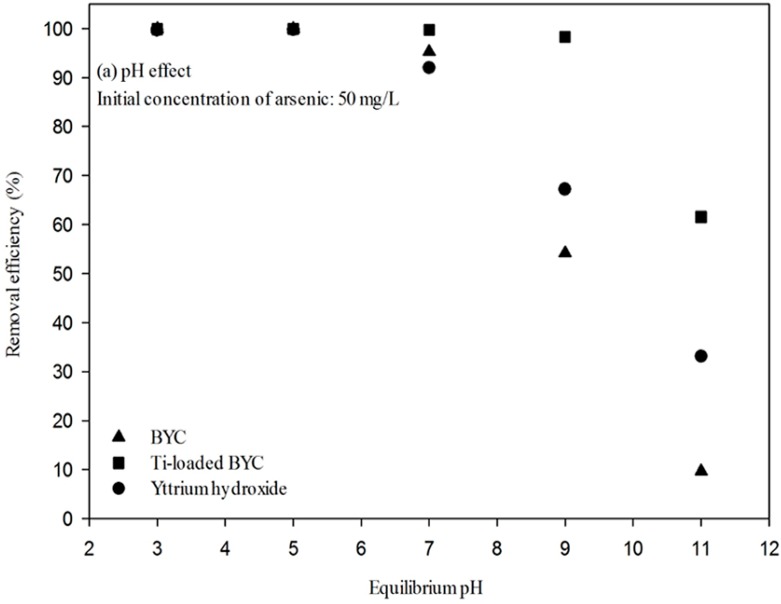

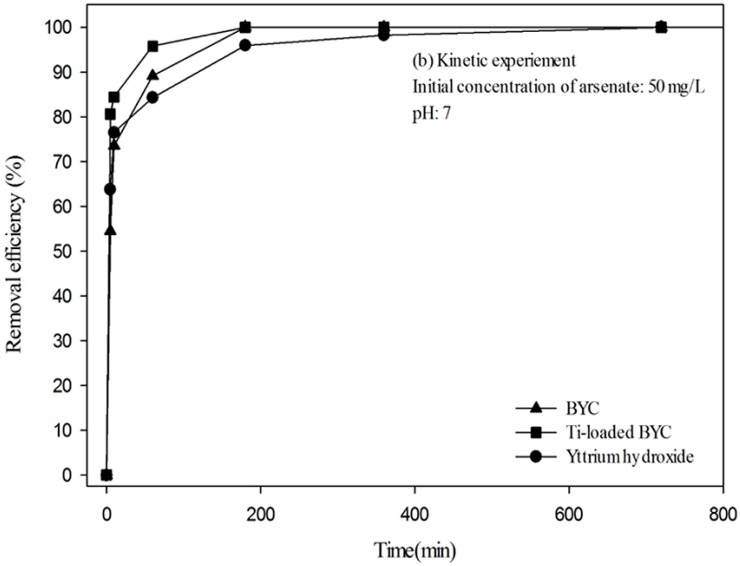
Adsorption efficiency by different pH (**a**) and kinetic results (**b**) of the arsenate adsorption (Initial concentration of arsenate: 50 mg/L; dose: 1 g/L, Reaction time: 24h; Temperature: 298K).

Figure 6a shows arsenate adsorption over the wide range of pH conditions; all cases show that the removal efficiency decreases with the increase in pH. Although the removal efficiency decreases, the Ti-loaded yttrium carbonate continues to display the best relative efficiency. In general, the adsorption rate of arsenate under basic conditions is lower than under acidic and neutral conditions because of the negative surface charge of the adsorbents above the point of zero charge [46]. 

Figure 6b presents the results of kinetic experiments. Among the candidates, Ti-loaded BYC has the best adsorption rate; however, all candidates removed 70% of arsenate within 10 min. The reaction rate was then calculated using pseudo-first and -second order kinetic models [33] with the results summarized in Table 2. Both kinetic models revealed the similar correlation coefficient (R^2^: 0.978–0.996), and the data showed that the rate constants such as K_1_ and K_2_ of the Ti-loaded BYC are faster (K_1_: 0.304, K_2_: 0.382) for arsenate than for BYC (K_1_: 0.151, K_2_: 0.085) and yttrium hydroxide (K_1_: 0.197, K_2_: 0.072). Therefore, the Ti-loaded BYC was deemed to be more favorable for arsenic removal in terms of reaction rate, which can be also explained by the modification of surface properties.

**Table 2:** Pseudo first order and second order kinetic constant of BYC, Ti-loaded BYC and yttrium hydroxide.

Adsorbents	Pseudo First Order Kinetic Model			Pseudo Second Order Kinetic Model		
	qe, cal (mg/g)	K_1_ (min^−1^)	R^2^	qe, cal (mg/g)	K_2_ (g/mg·min)	R^2^
BYC	97.67	0.151	0.993	58.41	0.085	0.978
Ti-loaded BYC	98.04	0.304	0.992	78.63	0.382	0.996
Yttrium hydroxide	95.24	0.197	0.986	62.52	0.072	0.993

**Figure 7:**
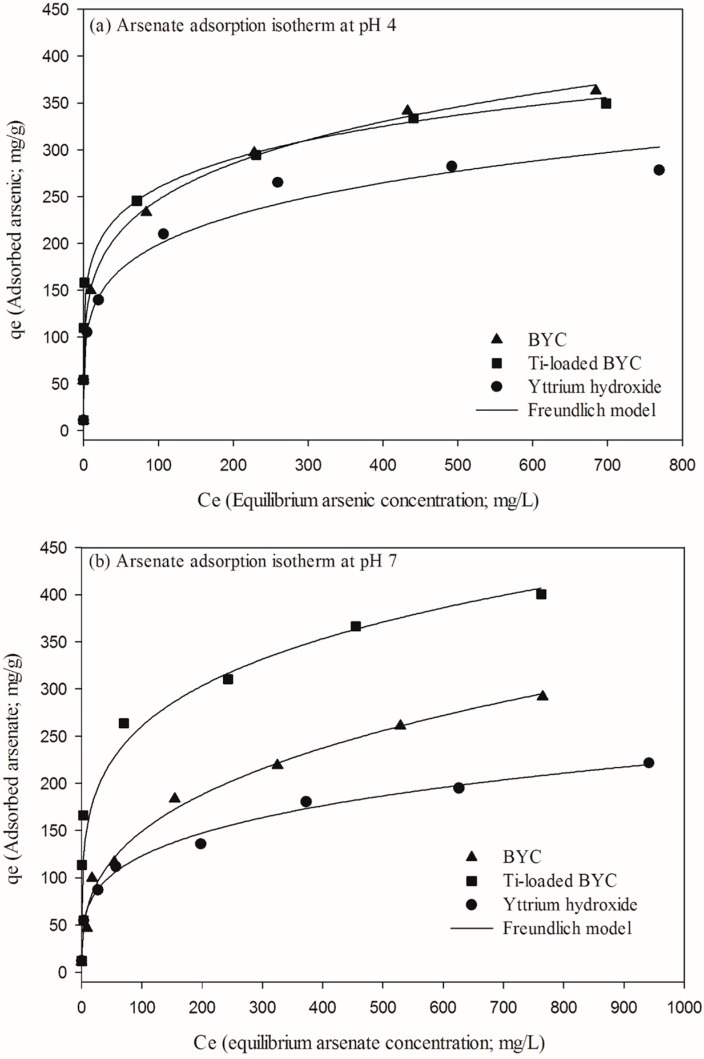

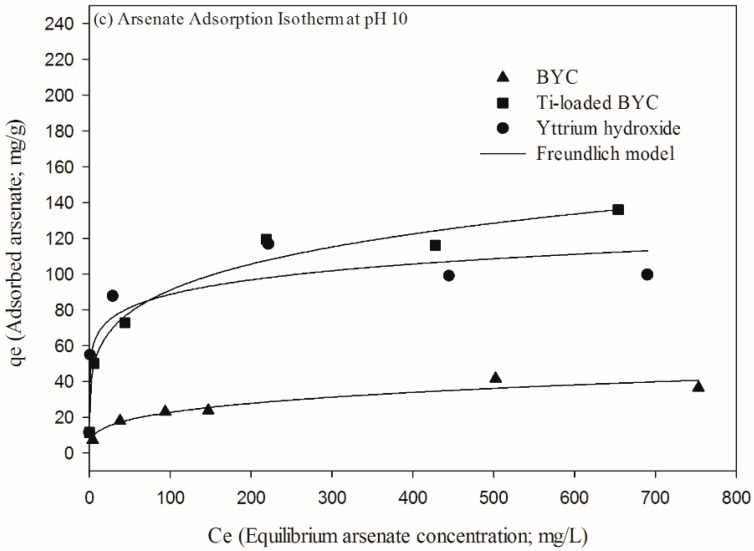
Adsorption isotherm at pH 4(**a**), pH 7(**b**) and pH 10(**c**) by the adsorbents (dose: 1g/L, Temperature: 298K, reaction time: 24h).

Langmuir and Freundlich isotherm Equations were applied to the results of isotherm experiments. The fitted data for the Langmuir and Freundlich models are summarized in Table 3 and Figure 7 shows the adsorption isotherm model for the different pH values. Although the equilibrium concentration of arsenate in BYC was lower than those in other candidates, yttrium carbonate has the best adsorption capacity in the fitted data, except at pH 10. The maximum adsorption capacity was 111.6 and 228.8 mg/g at pH 4 and 7, respectively. Most cases show that the Freundlich model displays a better fit than the Langmuir adsorption model; results of the isotherm model are summarized in Table 2. This result suggests that the adsorption between arsenate and yttrium based materials does not take place in the form of monolayer adsorption [47]. The adsorption affinity can be compared using the non-dimensional separation factor such as R:

(3) $$R=\frac{1}{1+K1C_{e}}$$

Adsorption is favorable if the R value is in the range of 0 to 1; if R is beyond 1, adsorption is unfavorable. The calculated R of Ti-loaded BYC is 0.573 when those of BYC and yttrium hydroxide are 0.568 and 0.476. The adsorption process for all materials is seen to be favorable for arsenate though the adsorption capacity of Ti-loaded BYC remains the most favorable for the materials tested.

**Table 3:** Isotherm constant of Langmuir and Freundlich for arsenate adsorption by the BYC, Ti-loaded BYC and yttrium hydroxide.

Adsorbents	pH Condition	Langmuir Isotherm			Freundlich Isotherm		
		Q_max_ (mg/g)	K (L/mg)	R^2^	K_F_ (mg/g)	1/N	R^2^
BYC	pH4	326.1	0.099	0.884	93.78	0.209	0.986
	pH 7	289.6	0.014	0.951	31.81	0.335	0.987
	pH 10	41.76	0.014	0.893	6.031	0.287	0.931
Ti-loaded BYC	pH4	303.5	1.451	0.886	123.1	0.162	0.964
	pH 7	348.5	0.152	0.841	94.77	0.219	0.938
	pH 10	128.6	0.031	0.836	30.44	0.229	0.957
Yttrium hydroxide	pH4	274.5	0.068	0.923	76.72	0.206	0.966
	pH 7	206.5	0.022	0.904	37.41	0.258	0.993
	pH 10	96.98	1.480	0.886	48.26	0.128	0.829

### 3.5. Effect of Coexisting Anionic Species and Desorption

**Figure 8:**
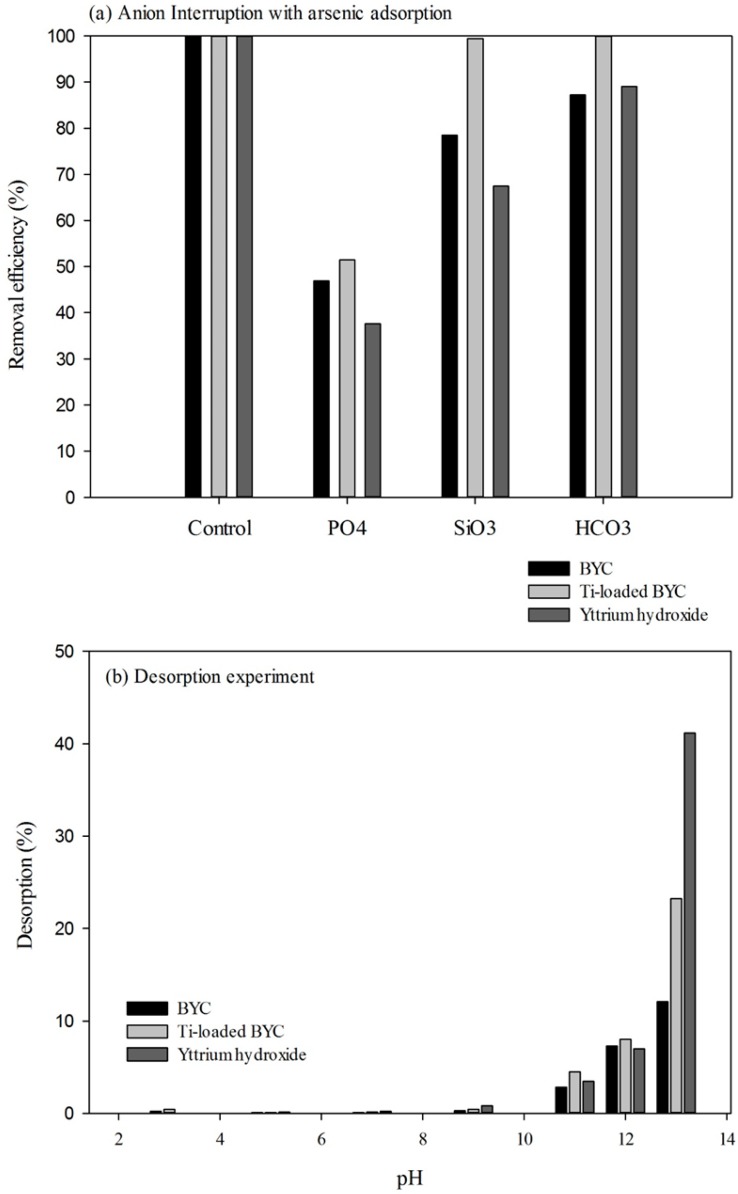
Anionic co-ion effect (**a**) and desorption results (**b**) by the adsorbents (condition (**a**); arsenic concentration: 10 mg/L, anion concentration: 10 mg/L, dose: 0.1 g/L, temperature: 298K, reaction time: 24h, condition (**b**); dose: 1 g/L, temperature: 298K, reaction time; 24h).

Figure 8a shows the effect of anions in arsenate adsorption, where interference can be seen for all coexisting anionic species. In particular, among the coexisting anionic species, phosphate was the greatest competitor with arsenate. The arsenate removal efficiencies of yttrium hydroxide, BYC and Ti-loaded BYC were 51.5%, 37.6%, and 46.9%, respectively. The interruption of arsenate adsorption by phosphate is general phenomenon, and several previous researchers have reported the adsorption behavior of arsenate and phosphate ion [48,49]; arsenate strongly competes with phosphate in the adsorption system. From the pH-pE diagram of arsenic, the dominant species of As(V) and phosphate in neutral pH region can be explained by HAsO_4_^2−^ and HPO_4_^2^. In case of HAsO_4_^2−^, the As–O bonding length is 0.1654–0.1671 and As–O bond in As–OH is 0.1742 nm. However, the length of P–O bond is 0.1510 to 0.1564 nm and the P–O bond in P–OH is 0.1551–0.1564 nm [50]. The ionic radii of phosphate (0.17 Å) are also smaller than that of arsenate (0.355 Å), and crystallographic size and ionic radii of the phosphate ion are also smaller than those of arsenate ion. Accordingly the phosphate ion (HPO_4_^2−^) has the high charge density and possibility than arsenate ion (HAsO_4_^2−^) [11,51]. Consequently, the sorption of arsenate ion can be interfered by the presence of phosphate ion. In these adsorbents, Ti-loaded BYC is seen to be less affected by anions, whereas yttrium hydroxide is strongly affected by the presence of anions. The Ti-loaded BYC may be the effective adsorbent even though the adsorption efficiency decreased by the effect of competitive anions. Figure 8b presents the adsorption stability in which all samples show stable adsorption for arsenate under various pH conditions except extremely basic condition (pH > 12). In the consideration of the adsorption affinity using the Langmuir equation, all materials were found to be favorable for arsenate adsorption. Arsenate adsorption using an yttrium-based material has high affinity, with Ti-loaded BYC displaying better affinity in these candidates than raw BYC and yttrium hydroxide due to its surface modification. The regeneration test demonstrates that the removal capacity of Ti-loaded BYC is 80 mg/g (25%) in the second adsorption cycle, however the third adsorption cycle showed the significant decrease (about 10 mg/g). Although the regeneration rate Ti-loaded BYC is limited at two times, it can be effectively used for the long-term removal system by its high removal capacity with economic feasibility. 

Even though the cost of yttrium compound is at least two times higher than those of iron and aluminum adsorbent, it may be applied to adsorption system since the removal capacity of yttrium compound is more five times higher (>300 mg/g) than iron and aluminum oxide (5–60 mg/g). Therefore, by using an appropriate granulation and coating method the yttrium compound can be used in a long duration removal system as part of a fixed-bed filtration system, and should overcome the current limit of frequent replacement of adsorbents in arsenic removal systems.

## 4. Conclusions

In order to develop new adsorbents for arsenate removal, yttrium-based adsorbent (BYC and yttrium hydroxide) and modified yttrium-based adsorbent (Ti-loaded BYC) were successfully prepared by a coprecipitation method targeting their high arsenate adsorption capacity. Batch results shows that Ti-loaded BYC has enhanced adsorption capacity. The enhanced adsorption capacity of Ti-loaded BYC is caused by increasing specific surface area (82 m^2^/g) with the formation of internal pores (10 to 100 nm) and increasing surface charge (PZC 8.4) than that of BYC (PZC 7.9). FT-IR results explained that the dominant arsenic adsorption mechanism can be accounted for by the chemical adsorption process between carbonate (CO_3_) and arsenate (HAsO_4_^2−^) ion exchange in BYC and Ti-loaded BYC, whereas yttrium hydroxide has a co-precipitation arsenic adsorption mechanism. The kinetic data revealed that Ti-loaded BYC displayed faster reaction rate than BYC and yttrium hydroxide. The maximum adsorption capacity was calculated by Langmuir isotherm, which shows that the maximum adsorption capacity of Ti-loaded BYC is 348 mg/g, and BYC and yttrium hydroxide displayed values of 289.6 and 206.5 mg/g, respectively. Additionally, the Ti-loaded BYC is effective over the broadest pH range (pH 3–11) and it can be effectively applied to drinking water (pH 5.5–8.5). Although all adsorbents showed adsorption interruption by phosphate competition experiment, Ti-loaded BYC only showed moderate level of interruption (50%). Desorption and regeneration studies showed that the yttrium-based adsorbents have high affinity for the arsenate ion. In summary, Ti-loaded BYC demonstrated enhanced adsorption capacity and may be effectively applied in an arsenate removal system via additional application methods such as granulation with strong affinity for arsenate.
